# Robotics Utilization for Healthcare Digitization in Global COVID-19 Management

**DOI:** 10.3390/ijerph17113819

**Published:** 2020-05-28

**Authors:** Zeashan Hameed Khan, Afifa Siddique, Chang Won Lee

**Affiliations:** 1 Department of Mechatronics and Biomedical Engineering, Air University, Islamabad 44000, Pakistan; 2 Pakistan Institute of Medical Sciences (PIMS), Islamabad 44000, Pakistan; drafifa.one@gmail.com; 3 Healthcare MBA Track & School of Business, Hanyang University, Seoul 04763, Korea; leecw@hanyang.ac.kr

**Keywords:** medical robots, COVID-19, healthcare digitization, coronavirus pandemic

## Abstract

This paper describes the evolving role of robotics in healthcare and allied areas with special concerns relating to the management and control of the spread of the novel coronavirus disease 2019 (COVID-19). The prime utilization of such robots is to minimize person-to-person contact and to ensure cleaning, sterilization and support in hospitals and similar facilities such as quarantine. This will result in minimizing the life threat to medical staff and doctors taking an active role in the management of theCOVID-19 pandemic. The intention of the present research is to highlight the importance of medical robotics in general and then to connect its utilization with the perspective of COVID-19 management so that the hospital management can direct themselves to maximize the use of medical robots for various medical procedures. This is despite the popularity of telemedicine, which is also effective in similar situations. In essence, the recent achievement of the Korean and Chinese health sectors in obtaining active control of the COVID-19 pandemic was not possible without the use of state of the art medical technology.

## 1. Introduction

**Figure 1:**
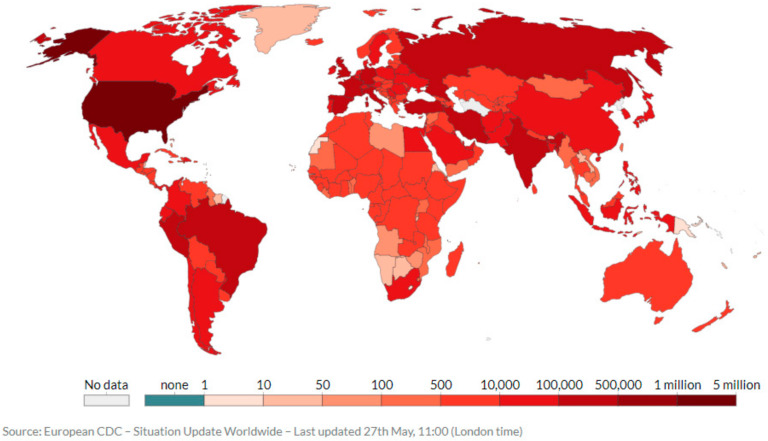
Total worldwide deaths due to COVID-19 per million people (as on May 27, 2020) [4].

The World Health Organization (WHO) on January 30, 2020 publicly declared the COVID-19 pandemic as a “global emergency” because of the rapidity at which it had spread worldwide [1]. The virus has shaken worldwide economies leading to a stock market crash in many countries. Since, the first bunch of cases identified in Wuhan City, China, in December 2019, the coronavirus pandemic has rapidly spread across China as well as over the borders, causing multiple incidents in nearly all countries of the world except Antarctica as shown in Figure 1. 

Despite the scarcity of publicly available data, scientists around the world have made progress in estimating the scale of the pandemic, the progression rate, and various transmission patterns of the disease [2]. Recently, clinical data confirmed that a significant portion of the COVID-19 patients show diminutive symptoms for the first four days, which illustrates the stealthy transmission potential of this contagious disease. Scientists have deliberated that COVID-19 is far more transmittable and lethal than the ordinary flu [3].

According to the WHO’s situational report 127 published on May 26, 2020, so far, 5,404,512 confirmed cases have been reported worldwide with 343,514 casualties [5]. The death rate is highest among older people compared to young ones, while male patients are more susceptible to risk compared to female patients in the same age group. 

**Figure 2:**
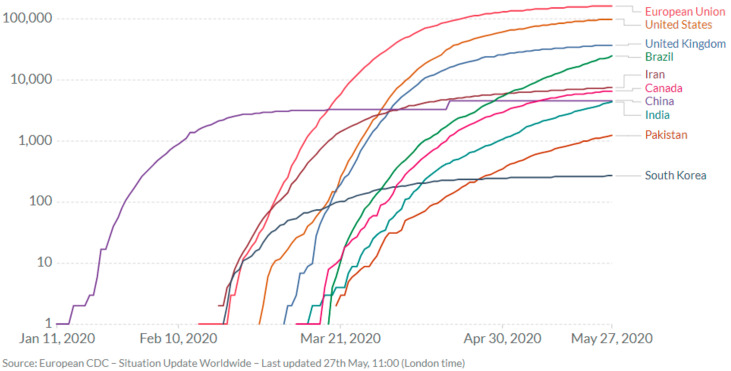
Total confirmed deaths due to COVID-19 on log scale (as on 27 May 2020) [4].

Patients with pre-existing cardiovascular diseases/hypertension, diabetes, cancer, and chronic respiratory disease have greater probability to pass away due to covid-19 complications compared to patients without comorbid conditions [4]. United States, China, Italy, Iran, Brazil, France, U.K, and Germany are so far the most affected countries of the world as shown in Figure 2. The routes of COVID-19 transmission can be pre-symptomatic, symptomatic or asymptomatic due to the highly contagious nature of the disease. Therefore, it is of utmost importance to use hand sanitizers, facemasks, and practice social distancing to avoid the viral infection, which can spread through sneezing, touching, and shaking hands. For the medical and healthcare community, the use of personal protective equipment (PPE) including N-95 facemasks and gloves for covering against the spread of corona-virus is mandatory for close monitoring of COVID-19 patients. Therefore, alternate technologies involving medical robots and tele-medicine systems are in focus in order to control the spread of infection to a large population [6]. 

Considering the current disastrous situation, robots are well suited for caring for the well-being of COVID-19 patients thus replacing or at least sharing the workload of the medical staff in hospitals under oversaturated conditions. A number of robotic systems are used for medical support in hospitals today [7]. In China, robots have been assigned multiple tasks to minimize the spread of COVID-19, such as utilizing them for cleaning and food preparation jobs in infected areas hazardous for humans. This study is one of the first studies, which highlights the importance of robotics in hospital and healthcare facilities specially concerned with the COVID-19 outbreak. 

The purpose of this study is to explore strategic healthcare digitization innovation through robotics utilization in terms of global COVID-19 management perspectives. This study will provide decision-makers and policy-makers with strategic insights in improving the healthcare quality in local and global disasters together with pandemic settings and other similar situations. 

This paper is organized as follows: Section 2 describes the key requirements of robots in the healthcare sector followed by the application-wise classification of robots in medicine and allied fields in Section 3. The viewpoint on COVID-19 management of incorporating robots is discussed in Section 4. Finally, the paper is concluded in Section 5. 

## 2. Requirements of Robots in Healthcare

**Figure 3:**
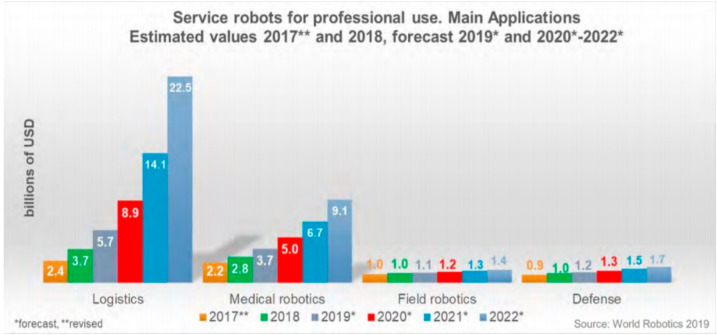
Increasing demand for medical robots in the world market [11].

The applications of robotics and automation in healthcare and allied areas is increasing day by day [8,9]. The International Federation of Robots (IFR) predicts an ever-increasing trend in the demand of medical robots within the coming years with an estimation of 9.1 billion USD market by 2022 as shown in Figure 3. Robots not only help physicians and medical staff to carry out complex and precise tasks but also lower their workload thus improving the efficiency of the overall healthcare facilities [10].

### 2.1. Kinematics and Dynamics

The requirement of kinematics and dynamics of a medical robot are application dependent. Serial as well as parallel robots are used in various tasks ranging from surgical and rehabilitation to service robots [12,13]. One such example of Parallel Kinematic Manipulators (PKM) is FlexPicker (ABB, Zurich, Switzerland) also known as “Delta” robot initially designed for surgical applications but also used in the food manufacturing industry extensively today [14]. Most of the service robots in hospitals are variants of mobile robots with a high payload capacity but with limited degrees of freedom (DOF). However, surgical robots with multi DOF are flexible, precise, and reliable systems offering similar performance to that of a well-trained human surgeon with a minimum error margin typically within millimeters [15,16].

### 2.2. Control and Dexterity

In order to carryout diverse tasks with high precision, reliability, and repeatability while minimizing the effects of external disturbances, the control of medical robotics is a challenging issue [17]. Moreover, while addressing the challenge of control and dexterity, designers need to allow sufficient degrees of freedom (DOF) for the end-effector to move in all the desired axes. Medical robots utilize state-of-the-art technology to carry out various tasks required for cleaning, sterilization, transporting, nursing, rehabilitation, and surgical applications. Adaptive robust embedded controllers are generally implemented for the control and navigation of such complex and agile robots [18].

### 2.3. Sterilization

Robots designed for use in healthcare and medicine have stringent cleaning requirements as they must be free of germs and microbes which can spread communicable and contagious diseases to other patients [19]. Most of the surgical end effectors are designed for single use only [16]. Service robots must be sterilized from time to time so that they do not become infective carriers [20]. Cooking robots have their separate protocol for cleaning, as they are washable after use.

### 2.4. Operator Safety

This is one of the prime requirement in medical robotics as the operator’s safety is very important while handling a robot in the hospital premises [21]. It should be safe enough for the operator, medical staff, and physician/surgeon as well as for the patient to have a robot in close proximity within the hospital without posing a threat to anybody. Surgical robots are required to meet the safety requirements as directed under standard IEC 80601-2-77 [22]. For rehabilitation robots, the basic safety and essential performance criteria is provided under standard IEC 80601-2-78.

### 2.5. Ease of Handling and Maintenance

Robots in hospitals are designed to be operated by medical technicians, medical doctors, and staff without engineering knowledge and troubleshooting skills. Therefore, the designers always need to ensure simple architecture, easy handling and quick maintenance for long-term usage of such machines [23]. Medical service robots help patients with prostheses, orthoses, hearing aids, and visual prostheses and thus require easy maintenance procedures [24].

### 2.6. Power Requirements

In order to operate medical robots, AC/DC power must be available without interruption so that these critical systems can work continuously. Since, medical facilities vary from large scale centrally located city hospitals to purpose-built field hospitals, various renewable energy sources are also utilized for reliable power solutions [25]. Wireless power transfer is also under development for mobile robots in hospitals to minimize the need for frequent charging [26].

### 2.7. Cost

Since, healthcare robotic solutions are needed at a large scale, they must be cost effective for easy installation and wide spread availability throughout the world, including developing nations [27]. High cost means that the scalability of these systems may not be feasible in most parts of the world. Surgical robotic systems are however, quite expensive solutions as they offer cutting edge technologies with integrated high definition video systems for tool guidance and maneuvering by the surgeon [28].

## 3. Classification of Robot Utilization in Healthcare 

Robots are mainly classified with various applications in healthcare and related fields. These classifications are broadly designated such as receptionist robot area, nurse robots in hospital area, ambulance robot area, telemedicine robot area, hospital serving robot area, cleaning robot area, spraying/disinfestation robot area, surgical robot area, radiologist robot area, rehabilitation robot area, food robot area, and outdoor delivery robot area. 

### 3.1. Receptionist Robots

**Figure 4:**
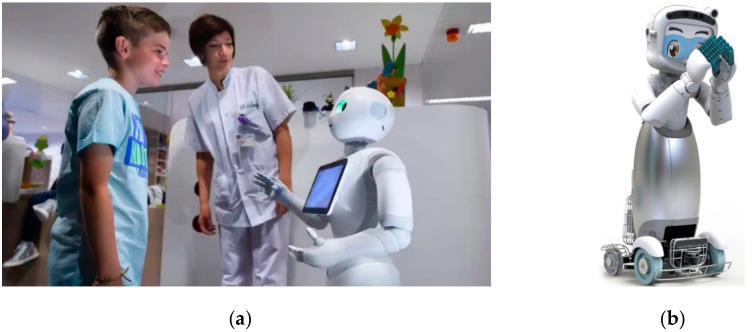
Receptionist robots in hospitals for patient assistance. (**a**) Pepper robot in a Belgian hospital [29]. (**b**) Dinsow 4 robot [30].

These types of robots are preferably used at a hospital’s reception to disseminate information about various units/sections of the hospital and guide patients and visitors as shown in Figure 4. They can handle a number of visitors without becoming tired and direct them to the physician of their choice [31]. They also are attractive to children coming to the hospital and amaze them by inducing pleasurable experiences and hence reducing their malaise symptoms.

### 3.2. Nurse Robots in Hospitals

**Figure 5:**
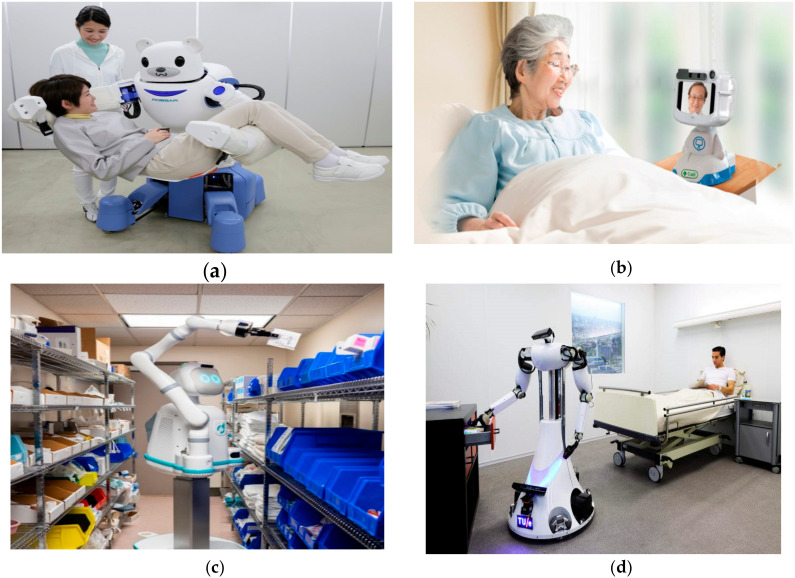
Nursing robots in hospital and at home for elderly care. (**a**) Robear—a robotic bear nurse to lift patients in Japan [33]. (**b**) Dinsow robot for elderly entertainment and face-to-face calls [34]. (**c**) Moxi—Nursing robot placing medicines in bins [35]. (**d**) Robot attendant for hospital care [36].

These robots are meant to assist doctors in the hospital in the same manner as that of human nurses. Nurse robots are commonly used in Japanese hospitals as Japan has the highest percentage of elderly (above 75 years) individuals among OCED countries. This poses a growing challenge for the medical facilities in the country. Without sufficient recruitment for elderly care, more Japanese citizens are socially bound in taking care of aging family members at home instead of doing a job [32]. In addition, the nursing and healthcare individuals undergo high stress and exhaustion due to patient load. Therefore, the Japanese government is looking towards technological solutions to take care of aged patients in the country as seen in Figure 5.

Humanoid nursing robots (HNRs) are likely to replace future nurses in Japanese healthcare facilities. Robots in nursing are becoming popular in offering services 24/24 and 7/7 with minimal costs [37]. In Japan, several nurse robots e.g., Paro (AIST, Toyama, Japan), Pepper (Softbank Robotics, Paris, France), and Dinsow (CT Asia Robotics, Bangkok, Thailand) are being used to assist elderly patients providing lifting as well as in therapeutic assistance. Each one of these robot plays a vital role in the Japanese healthcare system [38]. As an example, Dinsow has special features for Alzheimer’s patients. For example, it displays pictures of different people and asks the patient to match the face with a name to improve “dementia” symptoms [34]. However, the acceptance of service robots differs from country to country. Moreover, even if HNRs have values, some elderly patients have expressed the thought that human (staff) presence is also valuable as the machines are not able to replace humans entirely in the healthcare system. Table 1 presents a list of medical robots with applications.

### 3.3. Ambulance Robots

As per statistics, around 800,000 people per year suffer from cardiac arrest in the European Union (EU), out of which only 8% survive this emergency [39]. The main cause of this large number of victims is due to the comparatively sluggish response time of emergency services (typically 10 min) whereas brainstem death commences in just 4–6 min after severe cardiac arrest [40].

**Figure 6:**
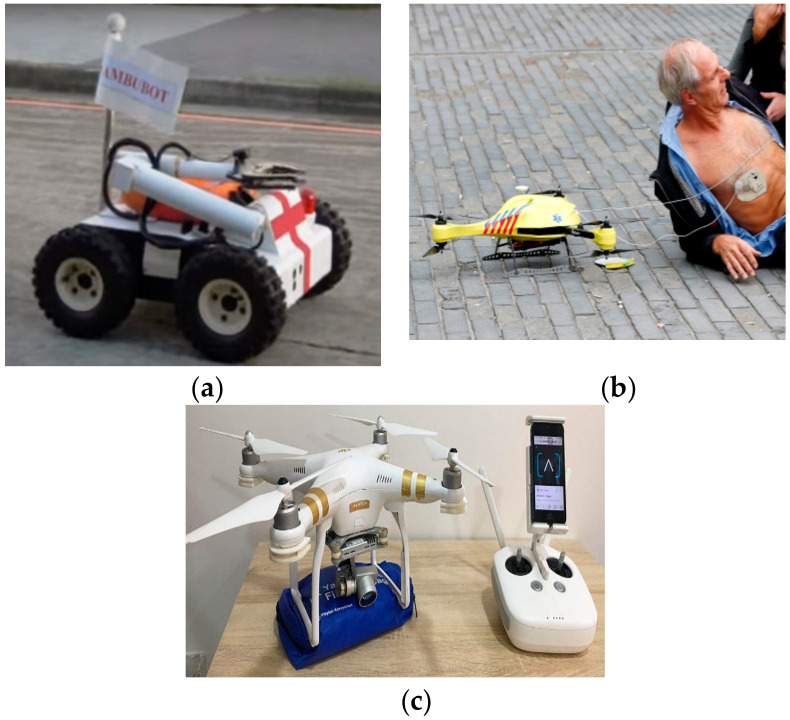
Ambulance robots. (**a**) Ambubot [39]. (**b**) Automated External Defibrillator (AED) for patient recovery [40]. (**c**) Drone carrying a first aid kit (blue) controlled by a smart phone [41].

The immediate medical aid after an accident is critical in order to prevent intensification of trauma. Thus, by speeding up emergency response, more lives can be saved as a result of fast recovery [41]. This is particularly factual for drowning, cardiac failure, shocks, and respiratory problems. Lifesaving strategies such as emergency medication, Cardiopulmonary Resuscitation (CPR), and Automated External Defibrillator (AED) aids can be designed lightweight and compact enough to be transported by a flying drone to the emergency site [39]. Such robots are useful in providing emergency treatment with minimum response time to a mobile or a distant patient as shown in Figure 6a,b. A lightweight miniature payload is developed for the Ambulance Drone (TU Delft, Netherlands), containing essential medical supplies for life support. The early prototype is meant to deliver an Automated Defibrillator (AED) as shown in Figure 6c [40]. 

### 3.4. Telemedicine Robots

Such robots are helpful in telemedicine applications where a remote doctor takes all the physiological parameters and diagnoses a disease using audiovisual aids [42]. Such systems are very helpful in the case of large-scale infectious epidemics in remote areas where hospitals and medical staff are not readily available. 

**Figure 7:**
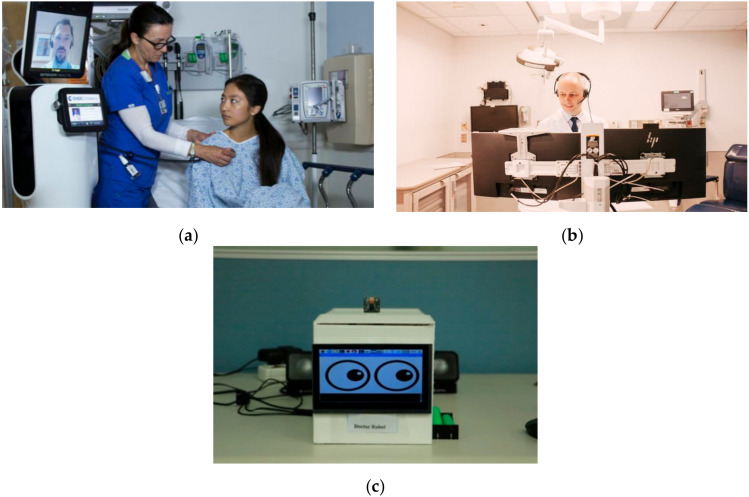
Telemedicine Robots for real-time remote patient assistance. **(a)** RP-VITA: FDA approved first autonomous telemedicine robot [43]. **(b)** Dr. Paul Casey, taking video calls at Rush University Medical Center [44]. **(c)** Doctor Robot for telemedicine [45].

Figure 7a shows RP-VITA (MedTech Boston, MA, USA), which is the first FDA-approved autonomous telemedicine robot. A doctor in a remote location can address the patients using real time audiovisual tele-conferencing as shown in Figure 7b. A telemedicine robot in Figure 7c is used for low-cost remote interface with the patients for diagnosis of ailments. 

### 3.5. Serving Robots in Hospital

**Figure 8:**
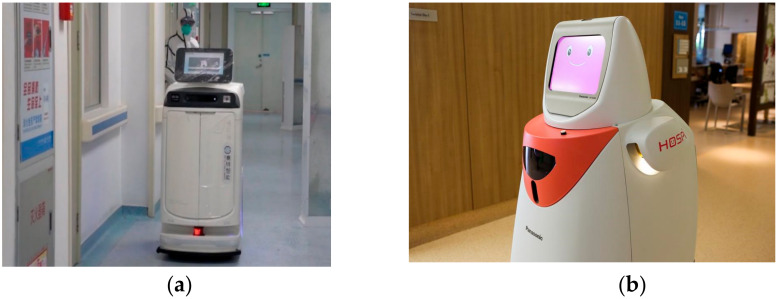

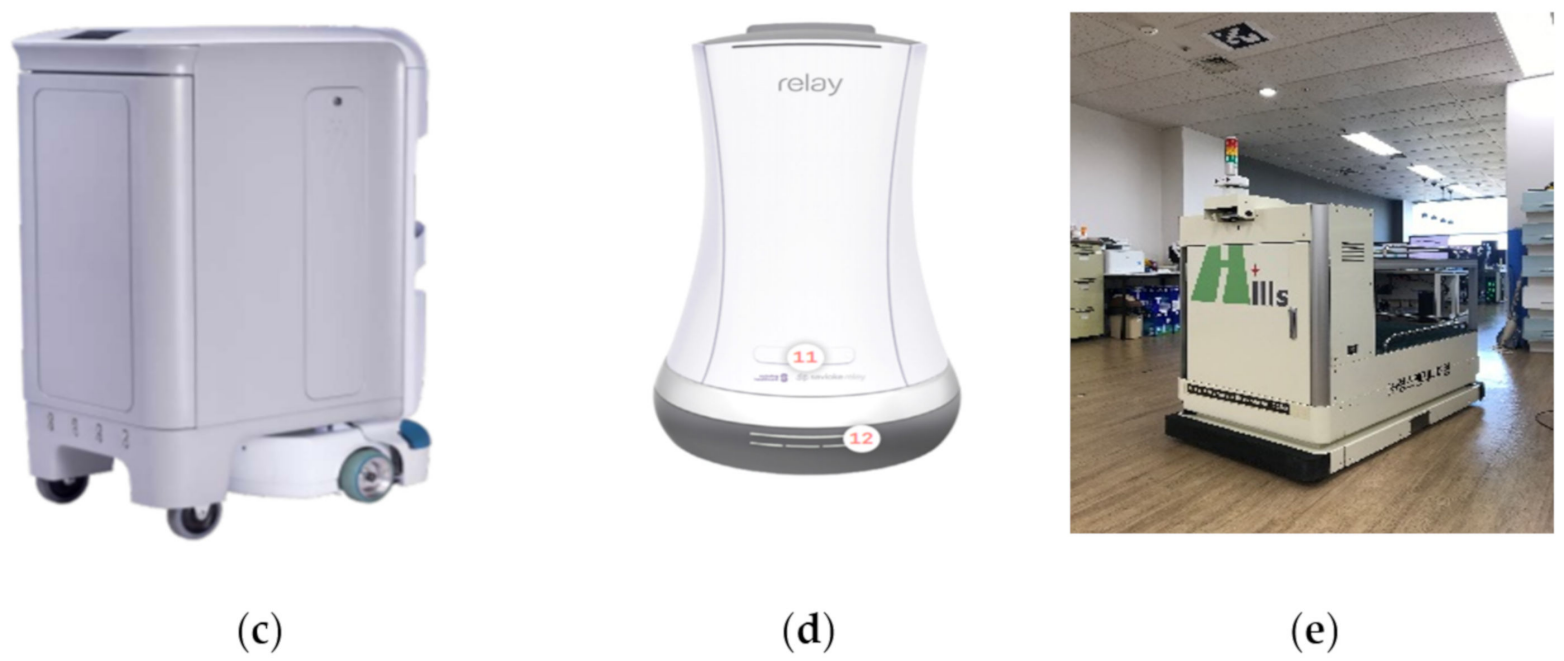
Indoor serving robots in hospitals. (**a**) Chinese hospitals using robots to deliver medicines in a patient’s room [48]. (**b**) Panasonic Autonomous Delivery Robots—HOSPI—deployed in a hospital in Singapore [49]. (**c**) TUG autonomous service robot [50]. (**d**) RELAY robot to deliver medicine [51]. (**e**) LoRobot L1 [52].

There are many tasks in hospitals where pushing and pulling of material is required. These heavy-duty tasks can be easily carried out by using serving robots [46]. Robots are also deployed to supply food to various patients residing in hospital [47]. They are used in the delivery of food and beverages, dispensing of drugs, removing of unclean laundry, delivery of fresh bed linen, and transportation of regular and contaminated waste etc. inside the hospital as shown in Figure 8 below.

### 3.6. Cleaning Robots

Robots are used in hospital cleaning using dry vacuum and/or mopping [53]. Cleaning robots for hospital environments seem to be able to provide the innovation, which the designers of non-industrial robot systems anticipated long ago. Such robots are an integral part of disinfecting hospitals to remove germs and pesticides. 

**Figure 9:**
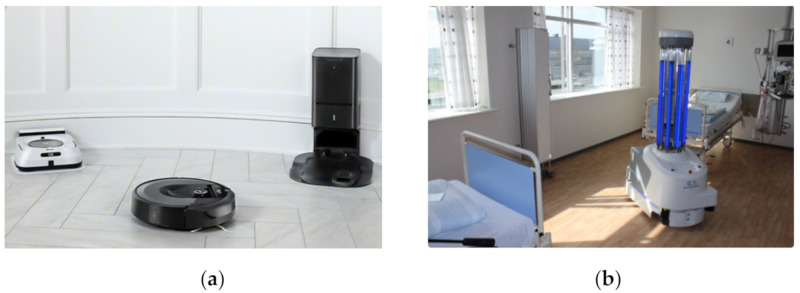

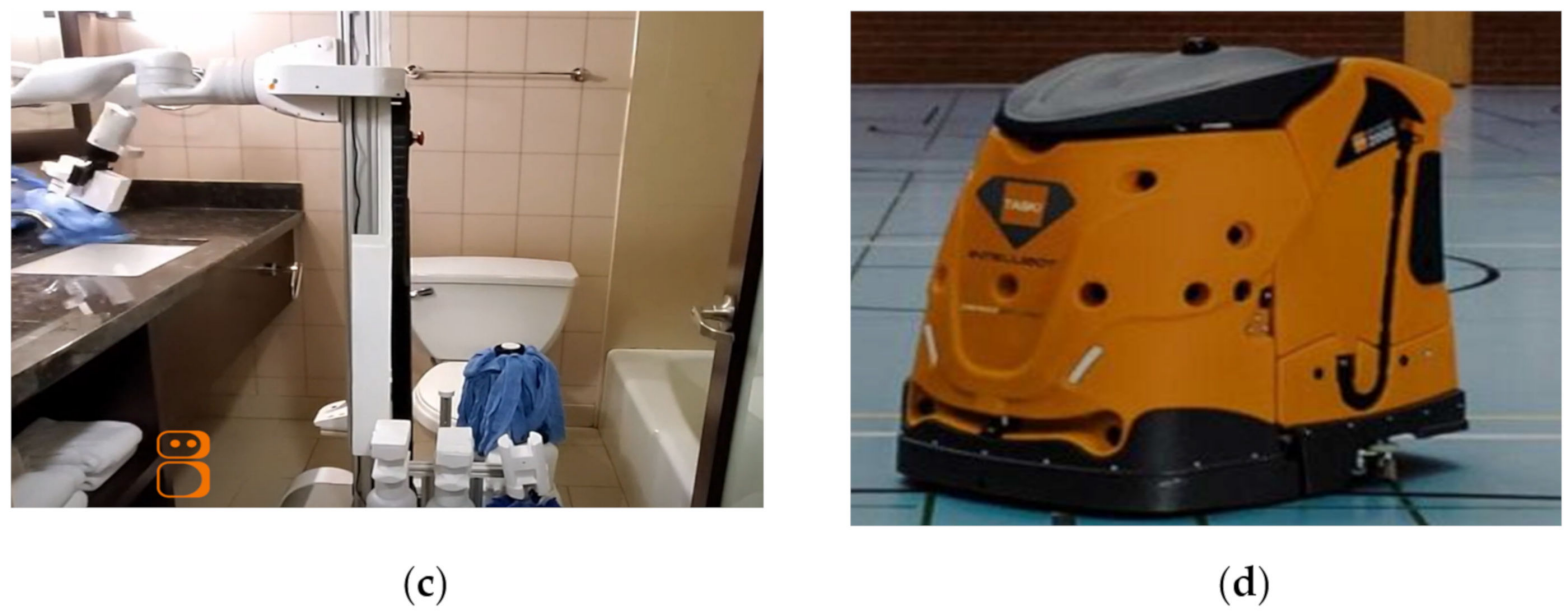
Cleaning and mopping robots in hospitals. (**a**) Roomba i7 cleaning robot [54]. (**b**) UVD robot for disinfecting hospital premises [55]. (**c**) Peanut robot for washroom cleaning [56]. (**d**) Swingobot 2000 cleaning robot [57].

Many such systems are depicted in Figure 9. Roomba cleaning robot (iRobot, Bedford, MA, USA) is an intelligent navigating vacuum pump for dry/wet mopping as shown in Figure 9a. UVD robot (UVD Robots ApS, Odense, Denmark) is a ultra-violet radiation based device used to disinfect hospital premises from microbes as presented in Figure 9b. Peanut robot (San Francisco, USA) in Figure 9c is used to clean washrooms of hospitals by using a highly dynamic robotic gripper and sensing system. Swingobot 2000 (TASKI, South Carolina, USA) is a heavy-duty cleaning robot for cleaning hospital floors autonomously as shown in Figure 9d.

### 3.7. Spraying/Disinfestation Robots

**Figure 10:**
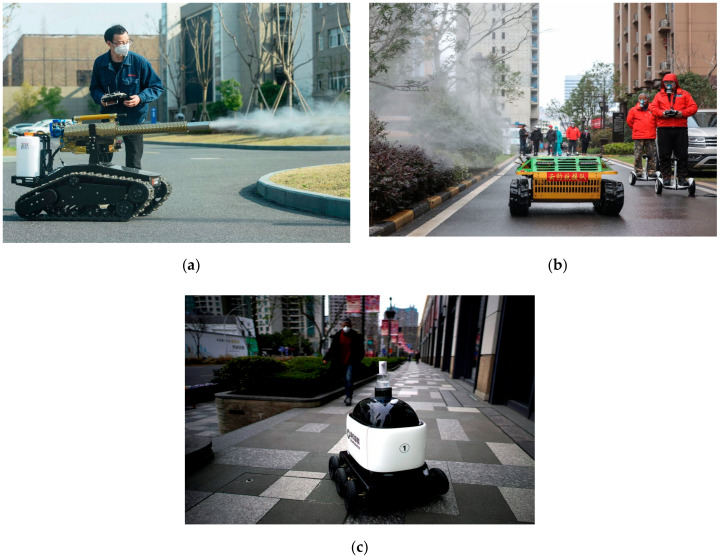
Spraying and sanitizing robots in residential areas of China [58]. (**a**) Remote control disinfecting mobile robot in Hangzhou, China. (**b**) Spraying robots to disinfect large residential areas in China. (**c**) A hand sanitizer-dispensing robot in Shanghai.

Such robots are widely used in spraying antiseptic mixtures over large outdoor areas e.g., residential centers of the city. These robots are remotely controlled to avoid hazardous contact with the disinfectant spray. Figure 10a,b shows such scenarios where sanitary workers on scooters control the robot in order to disinfect the surroundings.

Autonomously guided hand sanitizer dispensing robots are designed to alleviate infections on human hands and faces. Such alcohol-based sanitizers remove bacteria, viruses, and other microbes to prevent the spread of contagious diseases among large populations as shown in Figure 10c.

### 3.8. Surgical Robots

**Figure 11:**
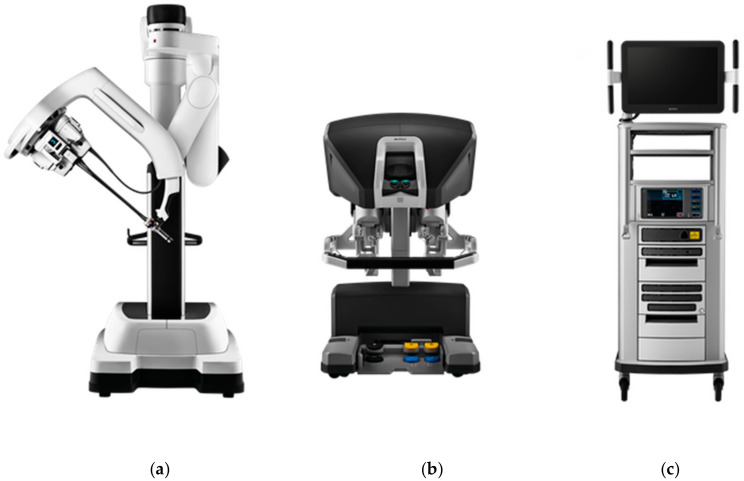
Da Vinci robotic surgical system [60]. (**a**) Patient cart. (**b**) Surgeon console. (**c**) Vision cart.

Surgical robots offer minimally invasive surgery (MIS) with precision and accuracy compared to human surgeons. Many tele-operators have been designed for remote surgeries [59]. The Da Vinci robotic surgical system from Intuitive Surgical Inc.is one such popular example as shown in Figure 11.

**Figure 12:**
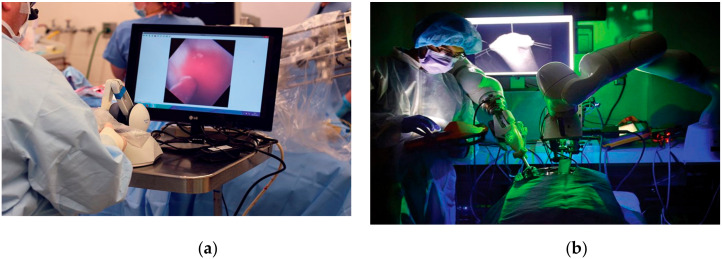
Real time surgery via HD vision using Da Vinci robotic surgical system [61]. (**a**) Surgeon remote manipulation. (**b**) Patient being operated by robotic hands.

Nowadays, fourth-generation Da Vinci surgical systems (Intuitive Surgical, California, USA) with complex machines but simple movements continue to advance MIS across a wide spectrum of surgical techniques. This offers upgradable design with flexible configurations and a dependable interface for surgeons using Da Vinci systems as shown in Figure 12. Standardization of instruments and components help hospitals to manage inventories and improve efficiency [60].

**Figure 13:**
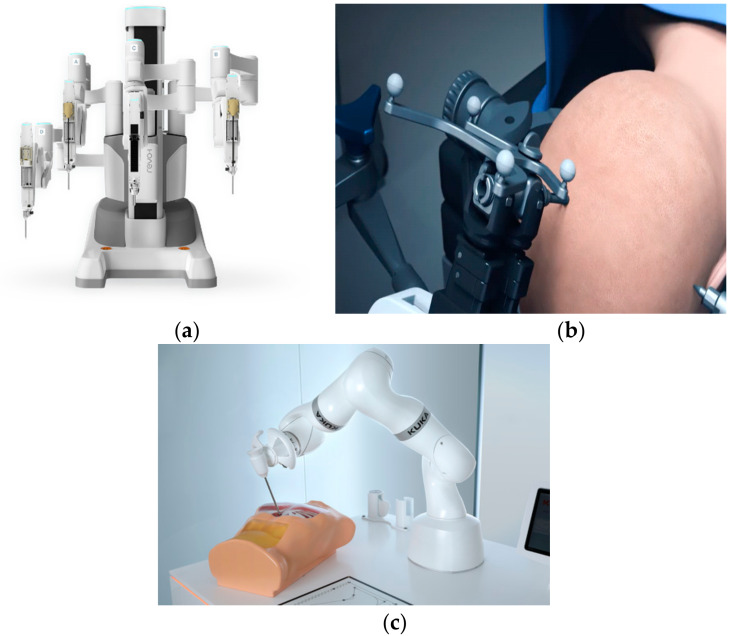
Specialized robots for general, spine and cranial biopsy and surgery. (**a**) Revo-i surgical robot [62]. (**b**) STEALTH AUTOGUIDE: Cranial Robotic Guidance Platform [63]. (**c**) KUKA LBR Med surgical robot [64].

Many other types of specialized surgical robots are in use for general endoscopic, cranial and spine surgery as well as for biopsy as shown in Figure 13a,b. Revo-I is termed the Korean version of the Da Vinci surgical system. KUKA LBR Med surgical robot (KUKA, Augsburg, Germany) is a high-performance serial robot for carrying out complex surgical procedures as shown in Figure 13c. 

The LBR Med is a sensitive seven-axis lightweight robot; it is very flexible and easy to integrate into medical products for numerous surgical interventions. It is perfectly suited to applications in medical technology due to its responsive sensors, inclusive safety provisions, hygiene-optimized surfaces, and a controller intended for direct collaboration with the human operator [64].

### 3.9. Radiologist Robots

**Figure 14:**
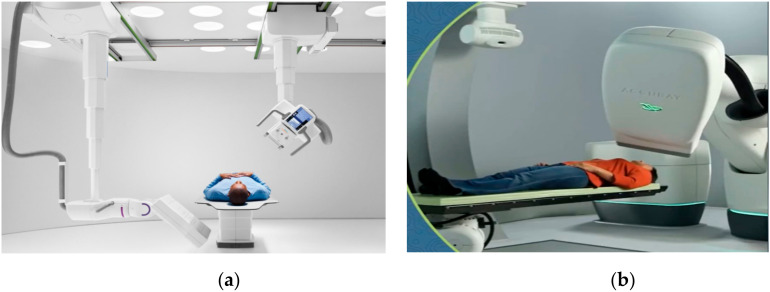
Specialized robots for spine and cranial biopsy and surgery. (**a**) Multitom Rax: Twin Robotic X-ray from Siemens Healthineers [65]. (**b**) Cyberknife: Radiology Oncology Surgical robot [66].

Radiology is one of the key technologies where robots are installed on special demand due to high level of radiations and safety issues for human operators. Twin Robotic X-ray (Siemens Healthineers, Henkestr, Germany) by Siemens is an innovation in radiology which offers fluoroscopy, angiography, and 3D imaging as shown in Figure 14a [65]. It performs a multitude of X-rays in just one room where the physician is able to see 3D images in real time as the robot moves instead of the patient. Conventional 2D X-rays usually fail to reveal fine hairline fractures in the bone and therefore require a computed tomography (CT) 3D image to confirm the diagnosis. With the Multitom Rax Twin Robotic X-ray system, a 3D image can be picked up conveniently on the same system, thus eliminating the need for a CT system.

Cyberknife precise robotic (Cyberknife Accuracy, Sunnyvale, USA) treatment is used for radiotherapy of cancer patients as shown in Figure 14b. It delivers stereotactic radiosurgery (SRS), and stereotactic body radiation therapy (SBRT), treatments anywhere in the body with high tech robotic precision and integrated real-time motion synchronization [66].

### 3.10. Rehabilitation Robots

**Figure 15:**
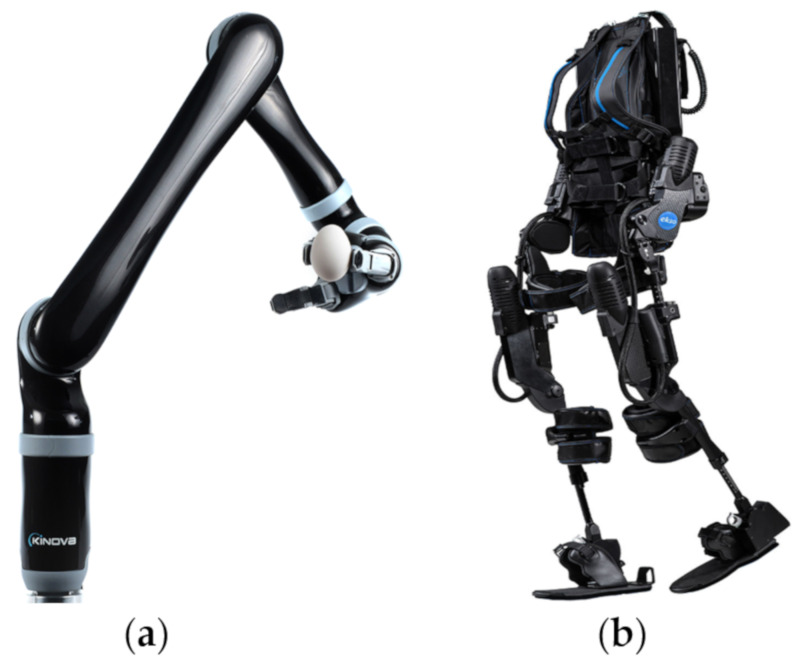
Rehabilitation and assistive robots. (**a**) Kinova assistive robotic arm [69]. (**b**) EksoNR exoskeleton [70].

These robots are helpful in rehabilitation of patients after an accident or stroke [27]. They are helpful in assisting and treating the disabled, elderly, and inconvenient conditions of people. These robots promote functional reorganization compensation and regeneration of the nervous system, effectively alleviating muscle atrophy [67]. As a result, rehabilitation physicians and staff are relaxed from overwhelming physical labor, thus optimizing the healthcare resources. Some examples of assistive and rehabilitation robots are shown in Figure 15 wherethe Kinova assistive robot (Kinova Robotics, Boisbriand QC, Canada) helps patients to pick and place objects with multi DOF performance and can be interfaced using brain computer interface (BCI). EksoNR (ekso bionics, Richmond, USA) is an exoskeleton for improved locomotion of disabled people [68].

### 3.11. Food Robots

**Figure 16:**
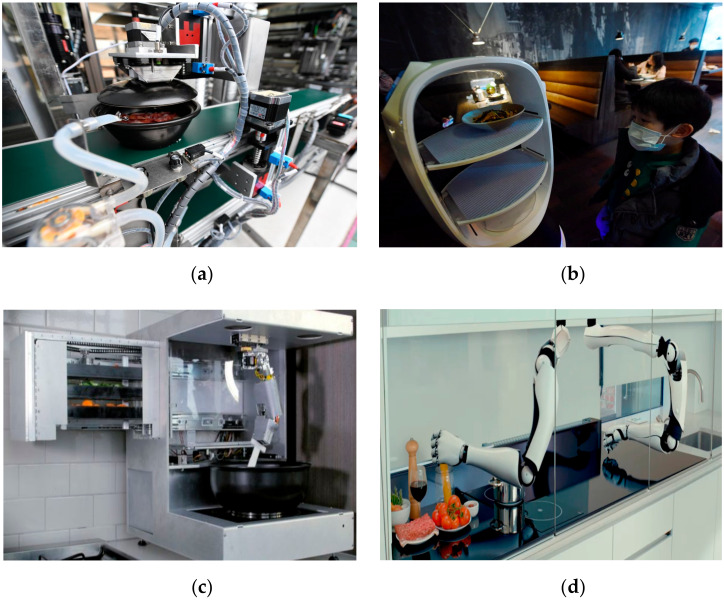
Food robots in a hospital’s kitchen for preparing and delivery. (**a**) Robot chef in a Chinese hospital [58]. (**b**) Food delivery robot in hospital [58]. (**c**) Cooki robot to prepare meals [71]. (**d**) Moley—World’s first robotic kitchen [72].

These robots are an integral part of a hospital’s kitchen and pantry in order to deliver high quality food as per hygienic standards [8]. From cooking to serving, various different types of automation and robotic systems have been designed by the roboticists. One such robot chef is used in Chinese hospitals as shown in Figure 16a. Serving robots to deliver food in hospital’s restaurants are shown in Figure 16b. Cooki (Sereneti Kitchen , Atlanta, Georgia, USA) and Moley (Moley Robotics, London, UK) are two different types of cooking robots with one and two robotic hands and are shown in Figure 16c,d respectively [71,72]. 

### 3.12. Outdoor Delivery Robots

**Figure 17:**
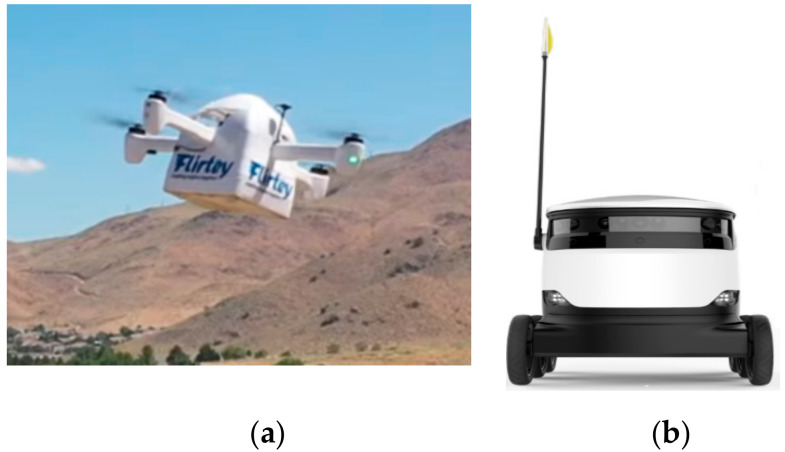
Outdoor delivery robots (ground based and aerial systems). (**a**) Flirtey drone robot for delivery of medicine/blood sample/food [73]. (**b**) Starship autonomous delivery robot [74].

Such robots are useful in transporting/delivering drugs and blood samples to/from the hospital. These fully autonomous robots can operate on the ground or in the air autonomously or with man-in-the-loop operation whereas an operator at a distance can remotely control them. Example systems include drone delivery robots by “Flirtey (Flirtey, Reno, Nevada, USA)” which aim to save lives and improve lifestyles by making delivery instant for everyone as shown in Figure 17a [73]. 

Starship robots (Starship Technologies, San Francisco, USA) are another example of ground based delivery robots that can carry less than 100 pound items within a 4-mile (6 km) radius as shown in Figure 17b. Items of interest include medicines, parcels, groceries and food, which are directly delivered from pharmacies and stores as per the order generated by customer requests via a mobile app. After placing the order, the robot’s entire journey and location is observed on a smartphone. Furthermore, to ensure secure delivery, an electromechanical lock is used to arm the cargo bay throughout the voyage. It is opened at the recipient end by using the smartphone app [74]. 

Table 2 presents a list of major medical robots with technical specifications involving their particular applications, weight, dimensions, nominal payload, operation duration, maximum speed, and the origin of the manufacturing company.

## 4. Viewpoint on COVID-19 Management

As shown in recent reports regarding COVID-19, most fatalities occur in old aged people especially with comorbidities as shown in Table 3 and Table 4 respectively. For the management of COVID-19 patients, guidelines have been established by centers for disease control and prevention (CDC). These guidelines are listed as follows [77,78]:

(a) Healthcare facilities should prioritize only urgent and emergency visits to fight against COVID-19. All elective and non-urgent admissions must be rescheduled.
(b) Preserving staff personal protective equipment (PPE) and patient care supplies for the safety of both is very important.
(c) Routine dental and eye-care visit must be postponed.
(d) All inpatient and outpatient elective surgical and procedural cases must be delayed to give priority to COVID-19 patients.
(e) Old age patients with underlying conditions are more vulnerable compared to young patients.
(f) Patients with underlying chronic medical conditions and pregnancy are at high risk due to this disease.
(g) Patients with mild clinical presentation may not initially require hospitalization.
(h) However, all patients with worsening signs and symptoms should be monitored closely with respect to the infection progress to the lower respiratory tract during the second week of illness.
(i) The decision to monitor a patient in the inpatient or outpatient setting is dependent on the clinical presentation.
(j) The estimated incubation period for COVID-19 is four days (interquartile range: 2–7 days) while some studies recommend a wider incubation of 2–14 days based on data from other coronaviruses (e.g., MERS-CoV, SARS-CoV).

### 4.1. Clinical Presentation

COVID-19 patients admitted to hospital have indicated symptoms e.g., fever, cough, shortness of breath, and fatigue as shown in Table 5. While fever is the dominating symptom of the patients admitted, other less commonly reported respiratory symptoms include sore throat, headache, cough with sputum etc. Prior to developing fever and lower respiratory tract symptoms, some patients reported gastrointestinal symptoms as well.

### 4.2. Diagnostic Testing

Specimens collected from the nose of patients are analyzed in labs for possible detection of COVID-19. Nasopharyngeal (NP) specimen is preferred for the detection of upper respiratory tract infection. Specimen from the lower respiratory tract including lung biopsy can also be helpful for patients presented with more severe disease. Serum analysis is helpful in monitoring antigens/antibodies count in COVID-19 patients. CT images of the chest have shown bilateral involvement in most patients [76].

Since, there is no currently available treatment for COVID-19, recommended infection prevention and control measures and supportive management of complications is the only possible clinical management. In some cases advanced organ support may be recommended as per need [78]. Few antiviral drugs e.g., Remdesivir and chloroquine have been shown to be affective against SARS-CoV-2 [80,81]. Moreover, many efforts are underway to produce a vaccine for COVID-19 virus. Patients affected by COVID-19 virus are required to isolate in hospitals or at home for two weeks.

### 4.3. Case Study—COVID-19 Wuhan, China

**Figure 18:**
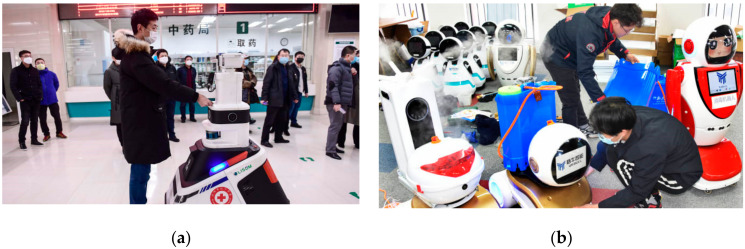
Robots in action during the COVID-19 epidemic in China [82]. (**a**) A patrol robot at a hospital in Shenyang in China’s northeastern Liaoning province checking temperatures of people. (**b**) Technicians adjusting disinfection robots in a technological company in Qingdao, east China’s Shandong Province.

**Figure 19:**
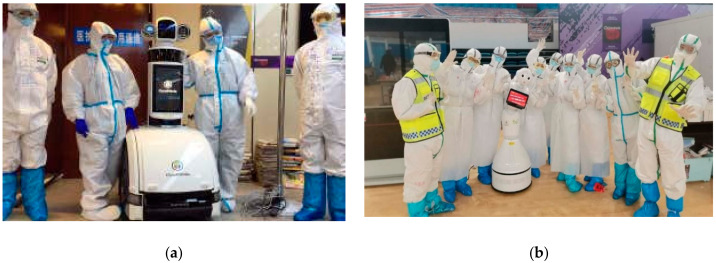
Reception robots in China during the COVID-19 outbreak. (**a**) Sterilization robot in Wuhan, China [83]. (**b**) Reception robot at a hospital ward in Wuhan, China [84].

The Chinese health ministry actively pursued the management of COVID-19 to avoid the blowout of the disease. In this pursuit, various robotic technologies were used to control and administer this epidemic as shown in Figure 18 and Figure 19.

**Figure 20:**
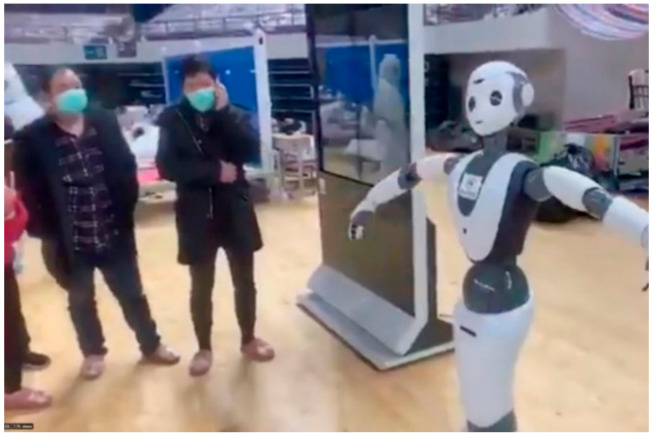
5G Cloud robots deployed in Chinese hospitals [85].

From service robots to disinfectant robots, hospitals frequently utilized technology to deal with a wide number of infected patients. Based on the technology-based solutions, very few cases were reported where infection was transmitted to the healthcare professionals. In China, such robots are effective in receiving the patients at the reception desk and monitoring their initial symptoms (such as flu and fever) and to disinfect them right at the entry point during the COVID-19 epidemic period. CloudMinds (USA) donated 5G Cloud Robots to various hospitals in Wuhan and Shanghai to help the Chinese healthcare community to fight against the coronavirus as shown in Figure 20.

It has been observed from expert reviews that the Chinese and Korean governments’ prompt reply in addressing the epidemic of COVID-19 was a successful attempt by using advanced measures and technology to manage the situation. These guidelines have been open for the world to take the same measures in order to stop COVID-19 fatalities. As seen from the data presented in Table 3 and Table 4 most of the serious cases arise in aged patients with comorbid conditions. For such patients, nursing and telemedicine robots are preferred. Moreover, in order to keep the environment clean, robots can help in disinfecting and sterilizing hospital premises and residential areas. A detailed mapping of these robotic technologies for COVID-19 management is depicted in Table 6.

## 5. Conclusions

This study presents a comprehensive overview of the robotics potential in medicine and allied areas with special relation to the control of the COVID-19 pandemic. Effective management of COVID-19 can significantly reduce the number of infected patients and casualties as witnessed in the case of the Chinese outbreak. Since, it has currently turned out to be a global challenge, technologically advanced countries can aid others by donating support equipment and robotic infrastructure to enable a good outcome in controlling this disease. This review substantiates that the introduction of medical robotics has significantly augmented the safety and quality of health management systems compared to manual systems due to healthcare digitization. Classification of medical robots is only done using application based categories to fit every aspect of hospital services ranging from cleaning robots to highly sophisticated surgical robots. Many opportunities are available in the design and operation of medical robots such as a cyber-physical system (CPS), power management using optimized algorithms and renewable sources, as well as fault tolerant control and dependable architectures for reliable and safe operation within the healthcare facilities.

## Figures and Tables

**Table 1:** List of prominent medical robots with application.

Robot	Make	Cleaning	Nursing	Pharmacy	Lab	Food Service	Waste Removal	Linen
Dinsow	CT Asia Robotics (Thailand)		✓					
Relay	Swisslog (Switzerland)			✓	✓	✓	✓	✓
TUG	Aethon (USA)		✓	✓	✓	✓	✓	✓
RP-VITA	iRobot (USA)		✓					
Roomba i7	iRobot (USA)	✓						
Moxi	Diligent Robots (USA)		✓	✓				
Ambubot	Thailand		✓	✓				
Drone Robot	TU Delft (Netherlands)		✓	✓				

**Table 2:** Comparison of various medical robots and their specifications.

Title	Application	Weight [kg]	Dimension [m^3^]	Nominal Payload [kg]	Operation duration [hr]	Max Speed [m/s]	Origin	Ref
RELAY	Service	40.8	0.021	4.5	4	0.7	Swisslog, Switzerland	[51]
TUG (T3 XL)	Service	635	1.034	-	10	0.76	Aethon, USA	[50]
HOSPI	Service	170	0.633	20	9	1.0	Panasonic, Singapore	[49]
RP-VITA	Telemedicine	79.37	0.565	-	4–5	Pan: 90 °/s Tilt: 60 °/s	iRobot, USA	[43]
Roomba i7	Cleaning	3.37	0.01	0.37	1.25	0.3	IRobot, USA	[54]
LG HOM-BOT	Cleaning	3.17	0.0086	0.5	1.75	0.35	LG, South Korea	[75]
KINOVA GEN3	Assistive	7.2	902 mm (max reach)	2.0	-	0.5	KINOVA, USA	[69]
EksoNR	Assistive	25	-	100	1	Variable	Ekso Bionics, USA	[70]
Mazor X	Spine Surgery	6.9	0.012	-	-	-	Medtronic, USA	[76]
Swingobot 2000	Cleaning	252	1.56	90	4	0.62	Diversey Inc, USA	[57]
Cyberknife	Radiosurgery	1267	1.01	240	-	-	Accuracy, USA	[66]
LoRobot L1	Service	200	0.84	100	8	1.2	Hills, South Korea	[52]

- refers to information not available or not applicable.

**Table 3:** Age related case fatalities of COVID-19 [79].

Patient Age	Case Fatality
30–39	0.2%
40–49	0.4%
50–59	1.3%
60–69	3.6%
70–79	8%
≥80	14.8%

**Table 4:** Comorbid case fatalities of COVID-19 [79].

Comorbidities	Case Fatality
Cardiovascular disease	10.5%
Diabetes	7%
Chronic respiratory failure	6%
Hypertension	6%
Cancer	6%

**Table 5:** COVID-19 patient’s reported symptoms [77].

Symptoms	Percentage
Fever	77–98%
Cough	46–82%
Myalgia or fatigue	11–52%
shortness of breath	3–31%

**Table 6:** Mapping of COVID-19 management steps using robotic solutions.

COVID-19 EDC Recommendation	Category	Prevention	Robotics Solution
Initial contact and assessment	Primary and emergency care	PPE, N95 face mask, goggles, gloves etc.	Robot doctor, Robot nurse, Ambulance robot
Hand hygiene	Personal hygiene	Sterilization	Sanitizer dispensing robot
Surface decontamination	Environmental hygiene	Use of hypochlorite or alcohol based disinfectants e.g., ethanol	Spraying robot for outdoor and UV robots for indoor disinfection
Patient Transport	Ambulance transfer	Surgical mask for the driver, PPE for the accompanying healthcare worker	Self-driving car (SDC) to carry patients
Hospital	Administration measures	PPE, N95 face mask, goggles, gloves etc.	Robot receptionist
	Patient management	as above	Tele-medicine Robot, lifting robot to shift patients from one place to another
	Pharmacy	as above	Medicine dispensing robot, drone robots
	Food services	as above	Robot chef, Food delivery robot
	House keeping	as above	Autonomous service robots
	Environmental Cleaning & waste management	as above	Cleaning/disinfection robots
Lab testing/Imaging	Blood test/sample collection/X-ray	as above	Sampling robot, Biopsy using surgical robot, SDC for sample collection, 3D X-ray and U/S robot
Management of the deceased	Administration measures	as above	Nursing robot for lifting, SDC for transportation to cemetery
Long term care facilities	Palliative Care	as above	Entertainment robots, tele-medicine robot, Nursing robot, Rehabilitation and Assistive robots
